# Pro-thrombotic condition in a woman with peripartum cardiomyopathy treated with bromocriptine and an Impella LP 2.5 heart pump

**DOI:** 10.1007/s00392-012-0494-5

**Published:** 2012-07-25

**Authors:** Marco R. Schroeter, Bernhard Unsöld, Karin Holke, Wolfgang Schillinger

**Affiliations:** 1 Herzzentrum, Abt. Kardiologie und Pneumologie, Universitätsmedizin Göttingen, Robert-Koch-Str. 40, 37099 Göttingen, Germany; 2 Abt. Pathologie, Universitätsmedizin Göttingen, Göttingen, Germany

Sirs:

Peripartum cardiomyopathy (PPCM) affects one in 300 to one in 100,000 pregnant patients, depending on cardiovascular risk factors (e.g. hypertension, diabetes, smoking) and gestational risk factors (e.g. advanced age of mother, number of children, malnutrition) [1]. The pathophysiology is poorly understood but inflammation/oxidative stress-mediated mechanisms and the nursing-hormone prolactin seem to be important. The therapy is supportive and an optimal medical therapy for heart failure is mandatory. The prognosis is favourable with the potential of complete recovery of left ventricular function within few months after diagnosis [2]. According to the severity of heart failure the implantation of a ventricular assist device should be considered in PPCM patients [3].

A Caucasian women aged 39 years was diagnosed with PPCM. Initially, the primipara presented in the Emergency Department of a local hospital with general fatigue, shortness of breath and oedematous ankles 4 days after normal delivery of a healthy baby. The symptoms started 2 days ago at home shortly after gynaecological discharge. She had no history of cardiopulmonary diseases and has taken no medication before pregnancy except l-thyroxin for thyroid hypofunction. She has obesity (BMI of 40 kg/m2) and previously smoked. During pregnancy, she has developed a gestational hypertension and diabetes that was treated with subcutaneous insulin therapy. The respiratory situation deteriorated rapidly, so that an intubation and mechanical ventilation had been necessary. In the following bronchoscopy spumous secretion could be aspirated. Due to highly cardiopulmonary instability she was transferred to our cardiological intensive care unit. In the thoracic X-ray and CT scan, a bilateral pneumonia and bilateral pleural effusions were detected (Fig. 1A). Pulmonary embolism was excluded. Laboratory findings indicated normal leucocyte count, haemoglobin 8.6 g/dL, normal platelets and slightly elevated PCT 0.3 μg/L. The initial blood cultures were sterile. The ECG showed sinus rhythm, normal repolarisation and depolarisation. Echocardiography indicated a dilated left ventricle (LVEDD 59 mm) with moderately reduced ejection fraction around 45 % (Fig. 1B), but with an acute severe functional mitral regurgitation due to a malcoaptation of mitral valve leaflets (Fig. 1C). The left atrium was dilated (46 mm), the right atrium and ventricle had normal size with an elevated systolic PAP of 46 mmHg. The N-terminal pro-BNP was 4,238 pg/mL indicating the severity of heart failure [4]. She was treated with antibiotics and catecholamine. We diagnosed a PPCM with cardiogenic shock after coronary artery disease was excluded [5] and we found no other possible cause for the cardiomyopathy. We treated the patient according to acute heart failure guidelines by inotropic agents (levosimendan 8 μg/min) and implanted an Impella LP 2.5 percutaneous micro-axial pump assist device (Abiomed Inc., Danvers, MA, USA) through the right femoral artery for left ventricular unloading (Fig. 1D + E). This resulted in a rapid and considerable reduction of mitral regurgitation (Fig. 1F). Moreover, a bromocriptine therapy (2.5 mg BID) was initiated, according to the position statement of European Society of Cardiology [6]. Intracardial thrombus was excluded by transesophageal echocardiography (TEE) and adequate heparin-based purge solution with 20 % glucose (500 IE heparin/h) for the Impella device and additional intravenous low dose heparin therapy (400 IE/h) was administered. The aPTT was in the normal range. Due to vaginal bleeding/ sanguineous lochia with the need of blood transfusions we could not further increase heparin doses. The gynaecologists could not detect a treatable bleeding source or other pathologies. However, within the following days the patient could be recompensated with reduced catecholamine therapy and respiratory pressures. After 5 days of treatment, a sudden increase in purge pressure was seen and the controller gave repeated catheter position alarms despite extensive repositioning attempts which finally lead to removal of the pump. After removal, a thrombus was visible within the pump and could be fully retrieved (Fig. 2A–C). We treated with full dose anticoagulation using low molecular weight heparin (with monitoring anti-factor Xa activity). The patient did not show any signs of neurological deficiency or persistent bleeding. On day 10, the patient could be extubated in stable cardiopulmonary condition, was transferred to the intermediate care unit on day 14 and was discharged on day 21. At discharge, the patient was in good clinical condition without signs of heart failure or neurological deficiency, and echocardiography indicated normal left ventricular function and dimensions (LVEDD 49 mm), and only mild mitral regurgitation.

**Fig. 1:**
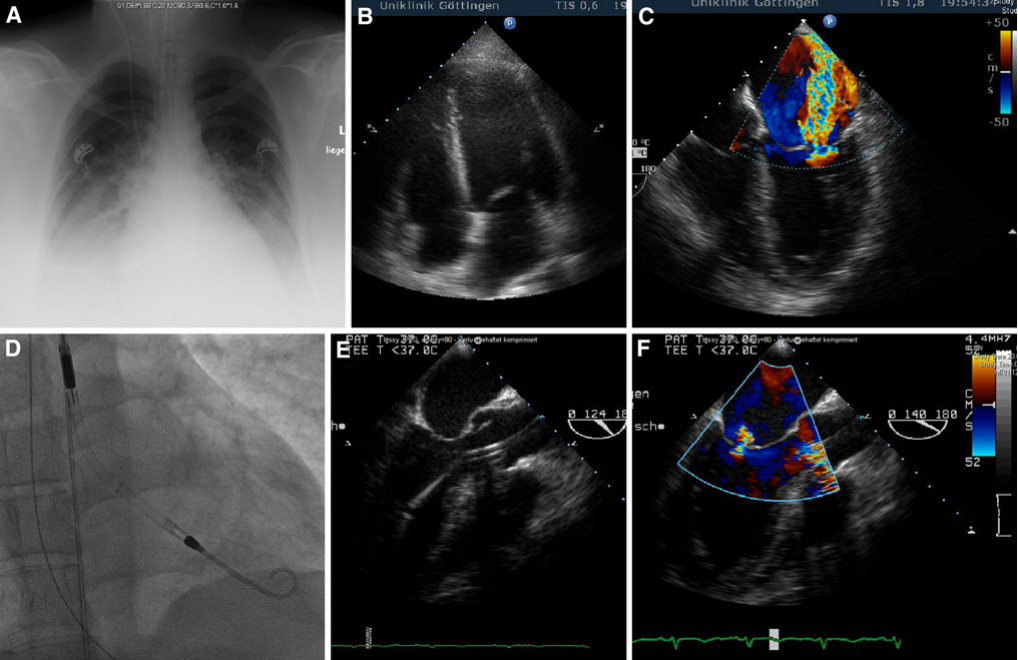
The patient with PPCM presented with bilateral infiltrates and pleural effusions, as shown in the thoracic X-ray (**a**). The transthoracic echocardiogram shows a dilated left ventricle (apical 4 chamber view; **b**) and the transesophageal echocardiogram (TEE) indicates an acute severe functional mitral regurgitation (ME 4 chamber view; **c**). An Impella LP 2.5 percutaneous micro-axial pump assist device was implanted under fluoroscopic control (**d**) and its correct position through the aortic valve could be verified by TEE (ME long axis view; **e**). After Impella pump implantation a reduction of mitral regurgitation could be shown (**f**). Philips SONOS 5500 with IPx-1 and HD11XE with S7-2 were used

**Fig. 2:**
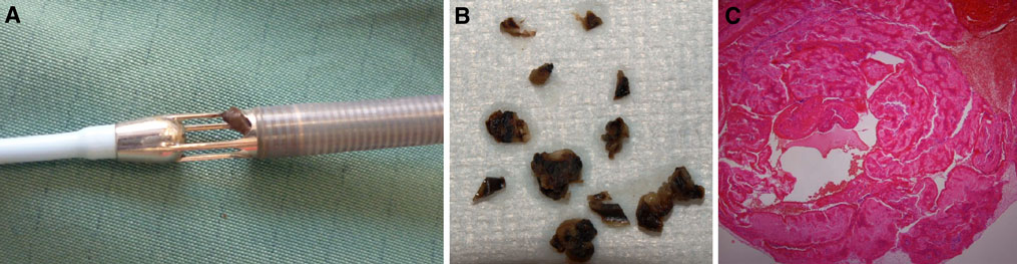
After removal of the Impella pump, a 3.4 × 0.6 cm grey-brown piece of tissue was visible within the pump (**a**) and could be retrieved. After fragmentation the histology with haematoxylin & eosin staining confirmed the presence of thrombotic material (**b**, **c**)

In our case, the Impella device was used because of left ventricular dilation with severe functional mitral regurgitation which facilitated cardiac recompensation by continuous ventricular unloading. Moreover, specific therapy with bromocriptin that suppresses the production of prolactin by dopamine D2 receptor agonism may be beneficial in PPCM [7]. Due to the risk of thrombo-embolic complications, including cerebral ischaemia and myocardial infarction in this period, anti-coagulation therapy is encouraged in PPCM patients those taking bromocriptine. Moreover, the PPCM itself could be associated with an increased thrombotic risk, due to increased procoagulatory activity in the peripartum phase [8] and reduced LV systolic function. In a small group of PPCM patients with highly reduced LVEF (<25 %) thrombo-embolism has not been observed under an adequate anti-coagulation therapy with warfarin [7]. The ESC guidelines indicate that in the context of reduced LV systolic function in PPCM, treatment with anticoagulation should be considered and it is recommended in patients with intracardiac thrombus detected by imaging or evidence of systemic embolism, as well as in patients with heart failure and paroxysmal or persistent atrial fibrillation [3]. In our case, the LVEF was only moderately reduced (with catecholamines), intracardiac thrombus was excluded and the patient persistently showed sinus rhythm. Although it is recommended to heparinise patients with Impella devices (ACT 160 s), studies indicated, that these patients do not need full dose systemic anticoagulation if a bleeding risk is present [9]. Yet, the presence of a thrombus on the Impella device which is uncommon when accurate anticoagulation is used in the purge solution [10] further emphasises the need for sufficient anticoagulation in PPCM patients.

This single case underlines the necessity of adequate anticoagulation therapy in PPCM with moderately reduced LV function and bromocriptine treatment, especially if ventricular assist devices are used, although a moderate bleeding risk is existent. Moreover, it demonstrates the beneficial effects of mechanical ventricular unloading as a supportive therapy in severe heart failure in PPCM.

## References

[1] Sliwa K, Fett J, Elkayam U (2006). Peripartum cardiomypathy. Lancet.

[2] Habli M, O’Brien T, Nowack E, Khoury S, Barton JR, Sibai B (2008). Peripartum cardiomyopathy: prognostic factors for long-term maternal outcome. Am J Obstet Gynecol.

[3] Regitz-Zagrosek V, Blomstrom Lundqvist C, Borghi C (2011). ESC Guidelines on the management of cardiovascular diseases during pregnancy: the Task Force on the Management of Cardiovascular Diseases during Pregnancy of the European Society of Cardiology (ESC). Eur Heart J.

[4] Sliwa K, Hilfiker-Kleiner D, Petrie MC (2010). Current state of knowledge on aetiology, diagnosis, management, and therapy of peripartum cardiomyopathy: a position statement from the Heart Failure Association of the European Society of Cardiology Working Group on peripartum cardiomyopathy. Eur J Heart Fail.

[5] Böhm M, Voors AA, Ketelslegers JM, Schirmer SH, Turgonyi E, Bramlage P, Zannad F (2011). Biomarkers: optimizing treatment guidance in heart failure. Clin Res Cardiol.

[6] Rademacher W, Lauten A, Lauten A, Ragoschke-Schumm A, Figulla HR (2010). Postpartum unmasking of a severe triple-vessel-disease with acute myocardial infarction. Clin Res Cardiol.

[7] Sliwa K, Blauwet L, Tibazarwa K, Libhaber E, Smedema JP, Becker A, McMurray J, Yamac H, Labidi S, Struman I, Hilfiker-Kleiner D (2010). Evaluation of bromocriptine in the treatment of acute severe peripartum cardiomyopathy: a proof-of-concept pilot study. Circulation.

[8] Brenner B (2004). Haemostatic changes in pregnancy. Thromb Res.

[9] Siegenthaler MP, Brehm K, Strecker T, Hanke T, Nötzold A, Olscheeski M, Weyand M, Sievers H, Beyersdorf F (2004). The Impella Recover microaxial left ventricular assist device reduces mortality for postcardiotomy failure: a three- center experience. J Thorac Cardiovasc Surg.

[10] Jurmann MJ, Siniawski H, Erb M, Drews T, Hetzer R (2004). Initial experience with miniature axial flow ventricular assist devices for postcardiotomy heart failure. Ann Thorac Surg.

